# Impact of body mass index, age and varicocele on reproductive hormone profile from elderly men

**DOI:** 10.1590/S1677-5538.IBJU.2014.0594

**Published:** 2016

**Authors:** K. G. R. Yamaçake, M. Cocuzza, F. C. M. Torricelli, B. C. Tiseo, R. Frati, G. C. Freire, A. A. Antunes, M. Srougi

**Affiliations:** 1 Divisão de Urologia da Universidade de São Paulo Faculdade de Medicina de São Paulo, Brasil; 2 Grupo no Centro de Reprodução Humana da Universidade de São Paulo Faculdade de Medicina de São Paulo, Brasil; 3 Faculdade de Medicina de São Paulo, Universidade de São Paulo, Brasil

**Keywords:** Aging, Receptors, FSH, Receptors, LH, Obesity, Gonadal Steroid Hormones, Varicocele

## Abstract

**Objectives:**

To study the impact of obesity, age and varicocele on sexual hormones fof adult and elderly men.

**Materials and Methods:**

875 men who were screened for prostate cancer were enrolled in this study. Data recorded comprised age, body mass index (BMI), serum levels of total testosterone (TT), free testosterone (FT), sex hormone-binding globulin (SHBG), luteinizing hormone (LH) and follicular stimulating hormone (FSH). Patients were divided in groups according to their BMI in underweight, normal weight, overweight and obese grades 1, 2 or 3. First, it was studied the association between age, BMI, and hormone profile. Then, clinical varicocele was evaluated in 298 patients to assess its correlation to the others parameters.

**Results:**

Obese patients had lower levels of TT, FT and SHBG (p<0.001) compared to underweight or normal weight patients. There were no differences in age (p=0.113), FSH serum levels (p=0.863) and LH serum levels (p=0.218) between obese and non-obese patients. Obese grade 3 had lower levels of TT and FT compared to obese grade 1 and 2 (p<0.05). There was no difference in the SHBG levels (p=0.120) among obese patients. There was no association between varicocele and BMI; and varicocele did not impact on testosterone or SHBG levels.

**Conclusions:**

Men with higher BMI have a lower serum level of TT, FT and SHBG. The presence of clinical varicocele as well as its grade has no impact on hormone profile in elderly men.

## INTRODUCTION

Obesity incidence has increased and it is getting higher with aging (1). This condition has strong correlation with cardiovascular diseases, hyperlipidemia, type 2 diabetes, osteoarthritis, hypertension, and even with some cancers (2, 3). Moreover, it has been associated with changes in the reproductive hormone profile, particularly in women (4, 5).

In men, total and bioavailable testosterone concentrations decrease with aging (6), leading to andropause. The actual prevalence of low-serum testosterone in aging men is not exactly known, but it is estimated at 25% (6, 7). The symptoms may include decreased libido, impaired erectile function, muscle weakness, increased adiposity, depressed mood, and decreased vitality.

Currently, some studies have indicated that varicocele may be a significant risk factor for androgen deficiency even for elderly men (8). There is evidence that varicocele has a negative impact on the Leydig cells function (9–11) and varicocelectomy can improve its function and increase the testosterone level (12, 13).

Therefore, there are data supporting the association between obesity, aging and varicocele with low reproductive hormone levels (14–16), however the interaction between these factors is still unclear. Herein, we aim to study the impact of obesity, age and varicocele on sexual hormones of adult and elderly men.

## MATERIALS AND METHODS

### Study design

We evaluated 907 men who were referred to our institution for prostate cancer screening in March 2012. Men with more than 50 years were eligible to participate. All patients were initially attended by a nurse responsible for assess their vital signs (temperature, blood pressure, heart beat rate, and glucose level). Patient's height and weight were also measured. All patients also filled out a questionnaire with their demographic data. Before medical visit, blood tests were collected and it could include the reproductive hormone profile if patients were in agreement. Eight hundred and seventy five men signed the consent term and were enrolled in this study. Institutional Review Board and Human Subjects Committee approved it.

The physical examination was performed by experienced urology assistants in all subjects following the same protocol. Testicular volume was measured with a Prader orchidometer (17). It uses a set of 12 models, ellipsoid shape, made of plastic with volumes of 1cm^3^, 2cm^3^, 3cm^3^, 4cm^3^, 5cm^3^, 6cm^3^, 8cm^3^, 10cm^3^, 12cm^3^, 15cm^3^, 20cm^3^ and 25cm^3^. Testicular volume was estimated by visual comparison to the set of models during physical examination.

All serum exams were performed at the same laboratory of our medical center using the same technique. All patients had hormone levels measured by a peripheral venous serum sample taken between 8:00am and 10:30am. Reproductive hormone profile included total testosterone (TT), free testosterone (FT), sex hormone binding globulin (SHBG), follicle-stimulating hormone (FSH), and luteinizing hormone (LH). TT and FT were measured by RIA assays (Diagnostic Systems Laboratories Inc., Webster, TX, USA). Their average sensitivities were 10ng/dL and 0.18pg/mL and the within-run and between-run coefficients of variation (CVs) were 8.3 and 7.7 and 6.2 and 8.9%, respectively. FSH and LH were measured by FIA (AutoDELFIA hLH, Wallac Oy, Turku, Finland) with sensitivity limit of 0.15mU/mL and CVs of 6.6 and 7.3%, respectively. The CV for SHBG was 5%.

Initially we studied the impact of patient's age and BMI, calculated by dividing weight by height-squared (kg/m^2^), on reproductive hormone profile. Patients were divided according to their age in four groups: under 50, 50 to 60, 60 to 70, and over 70 years. They were also classified according to their BMI in underweight (≤18.5kg/m^2^), normal weight (18.6–24.9kg/m^2^), overweight (25–29.9kg/m^2^), obese grade 1 (30–34.9kg/m^2^), obese grade 2 (35–40kg/m^2^), and obese grade 3 (≥40kg/m^2^). A correlation between age and total testosterone, free testosterone, SHBG and FSH, and LH was performed for each group. Thereafter, a correlation between BMI and total testosterone, free testosterone, SHBG and FSH, and LH was also carried out for each group. In addition, a correlation between age and BMI was also performed.

In addition, 298 subjects were evaluated for presence and grade of varicocele. The selection was randomly performed. The subgroup of patients that were examined for varicocele had similar mean age and BMI (60.5 years and 27.7kg/m^2^) when compared with the remaining patients. Physical examination was conducted in a warm and comfortable office with patient in standing position. Varicocele was classified, if present, according to the modified criteria of Dubin and Amelar (18) as follow: grade 1, dilation of spermatic cord palpable only while performing Valsalva maneuver; grade 2, dilatation of the spermatic cord palpable without the Valsalva maneuver; or grade 3, massive dilatation of the spermatic cord is visible without Valsalva maneuver. In cases of bilateral varicocele, the side with the high grade was used to final classification. In this subgroup of patients, correlation between BMI, age, testicular volume, hormone levels and varicocele (presence and grade) was performed.

### Statistical analysis

SPSS version 20.0 (Chicago, IL, USA) was used to analyze the collected data. All data were descriptive and based on frequencies. Comparison of continuous variables was carried out using Student's t test. Groups were compared with Kruskal-Wallis one-way analysis of variance. Significance was set at p<0.05.

## RESULTS

### Age and Body Mass Index

**Figure 1:**
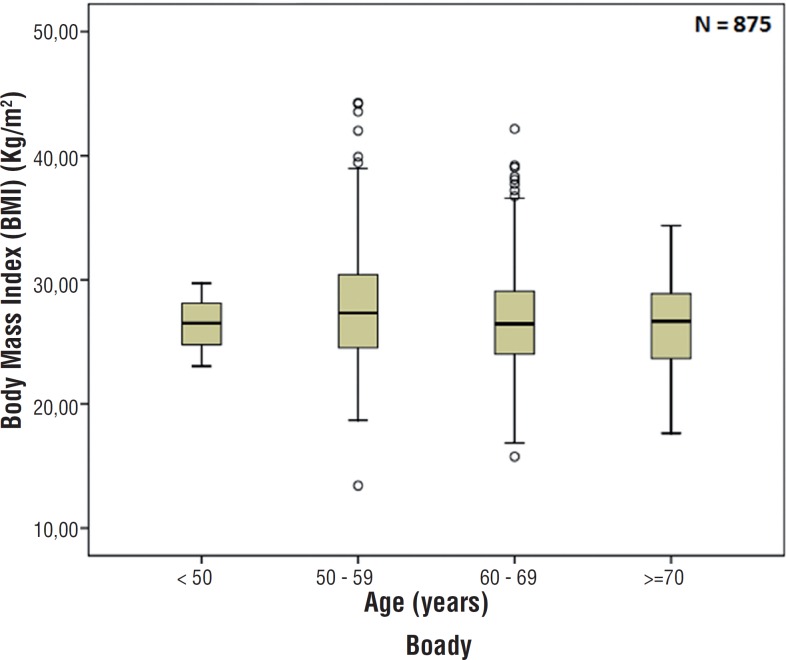
Association between age and body mass index (p=0.130).

**Figure 2:**
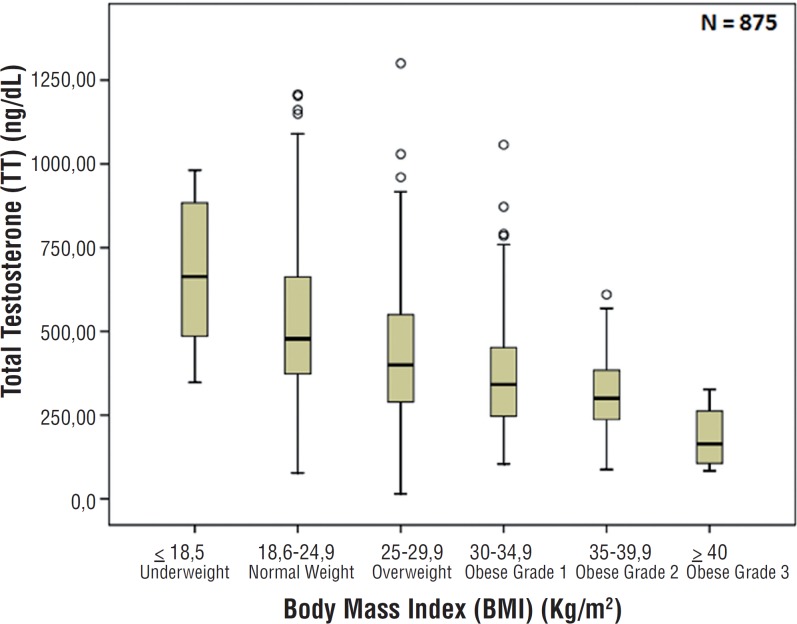
Association between body mass index and total testosterone (p<0.001).

**Figure 3:**
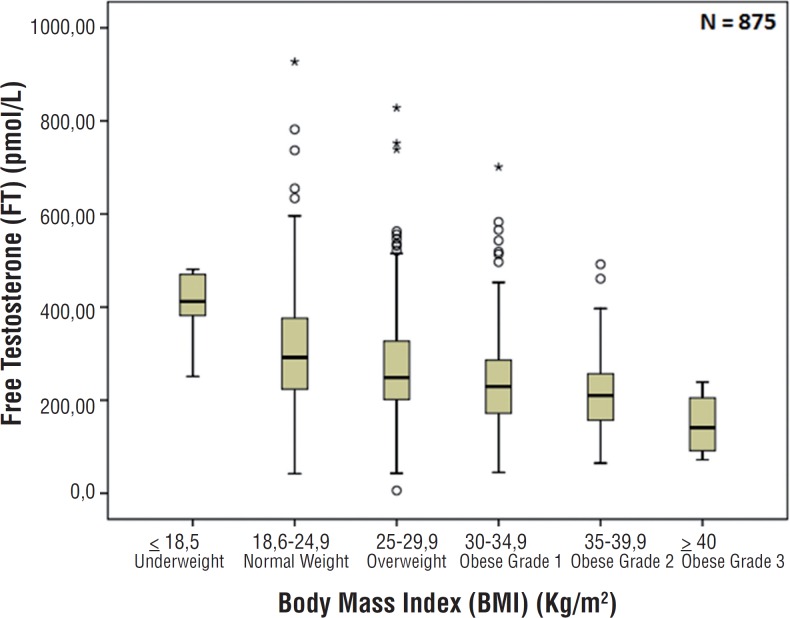
Association between body mass index and free testosterone (p<0.001).

**Figure 4:**
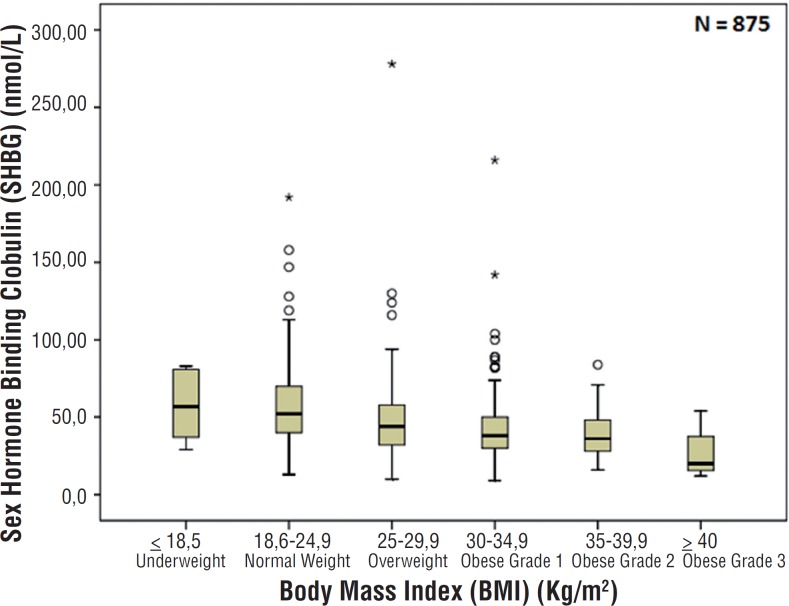
Association between body mass index and sex hormone binding globulin (p<0.001).

Demographic data and reproductive hormone profile are summarized in Table-1. There were no associations between age and TT, FT, SHBG, FSH and LH levels. There was also no association between age and BMI (p=0.130) as showed in Figure-1. Comparing BMI and reproductive hormone levels, it was observed that obese patients had lower hormone levels. Obese patients (grade 1, 2 and 3) had a significant lower level of TT, FT and SHBG (p<0.001) compared to underweight and normal weight patients–Figures 2, 3 and 4. However, there were no significant differences in the TT (p=0.178) and SHBG (p=0.933) levels from underweight and normal weight patients. There were also no differences in the TT (p=0.149) and FT (p=0.443) from obese grade 1 and 2. In addition, there was no difference regarding the SHBG levels from obese grade 1, 2 and 3 (p=0.120). FSH and LH levels had no difference between the groups in relation to BMI (p=0.863 and p=0.218; respectively).

**Table 1:** Demographic data and reproductive hormone profile.

	Population in study	Reference values
	875	-
Age (years)*	61.0±6.0 (46–87)	-
Weight (kg)*	79.0±14.0 (31–140)	-
Body Mass Index (Kg/m^2^)*	27.2±4.4 (13.4–44.3)	-
Total testosterone level (ng/dL)*	452.4±211.5 (15–1300)	271–965
Free testosterone level (pmol/L)*	282.3±126.7 (5–927)	131–640
SHBG level (nmol/L)*	49.6±24.4 (9–278)	12–75
LH level (IU/L)*	5.0±4.0 (0.8–71.4)	1.0–8.4
FSH level (IU/L)*	7.1±6.6 (1.0–70.8)	< 10.5

* Mean±SD (range)

### Varicocele

**Figure 5:**
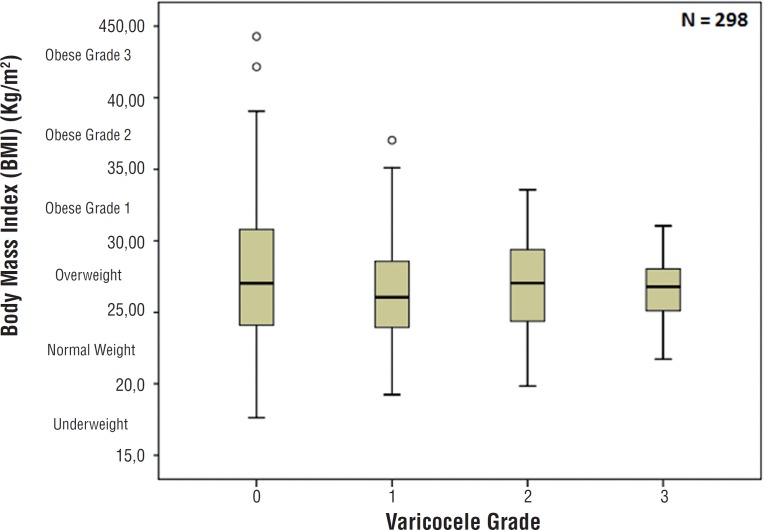
Association between total testosterone levels, presence and grade of varicocele (p=0.993).

The prevalence of varicocele was 36.3% (n=106). Thirty-five, 52, and 19 patients had grade 1, 2 and 3 varicocele, respectively. There was no significant association between the presence or grade of varicocele and sexual hormone levels Table-2. The median (Q1-Q3) of TT levels (ng/dL) in varicocele grade 1, 2 and 3 were: 413 (257–535), 392 (276.5–527.5) and 358 (280–493), respectively-Figure-5. Patients with or without varicocele had similar BMI (p=0.418). There was no significant association between grade of varicocele and BMI (p=0.993). There was no association between varicocele grade and testicular volume (p=0.419).

**Table 2:** Comparison between patients with and without varicocele.

	Reference value	Absence of varicocele	Varicocele group	p value
		192	106	
Age (years)*	-	60 [56–66] (46–76)	60 [56–66] (50–79)	0.516
Weight (kg)*	-	80 [69–90] (48–131)	80 [70–87] (48–116)	0.857
Body Mass Index (Kg/m^2^)*	-	27.0 [24.1–30.8] (17.6–44.3)	26.6 [24.5–29.1] (19.2–37.0)	0.418
Total testosterone level (ng/dL)*	271–965	388.5 [263.5–517.5] (118–1203)	395.0 [277.0–523.0] (15–1601)	0.973
Free testosterone level (pmol/L)*	131–640	243.5 [189.0–307.5] 91–828)	256.0 [180.0–332.0] (6–566)	0.677
SHBG (nmol/L) level*	12–75	42 [30–58] (10–158)	45 [32–56] (10–100)	0.674
LH level (IU/L)*	1.0–8.4	4.0 [2.7–5.6] (0.8–34.2)	3.8 [3.0–5.4] (1.8–29.9)	0.384
FSH level (IU/L)*	< 10.0	4.9 [3.2–7.2] (1.0–70.8)	5.2 [3.5–7.5] (1.0–64.9)	0.244

* Mean [Q1-Q3] (range)

## DISCUSSION

In this study we noted that obese patients have lower levels of some hormones such as TT, FT and SHBG compared to underweight and normal weight patients. There was not found any association between BMI and age in our population. Regarding varicocele analysis, the presence and grade of varicocele had no impact on TT and SHBG. Patients with varicocele compared to patients without varicocele were similar regarding age, BMI and hormone levels.

The behavior of sex hormone levels in middle-aged men has gain interest due to hormone replacement with steroids. Several large cross-sectional studies have come to consensus on a 1–2% annual decline in free or bioavailable testosterone (6). Despite strong evidences of the decline of sex hormone levels, our study did not demonstrated correlation between aging and hormone levels. Maybe it could be influenced by the smaller subset of patients older than 65 years and less than 40 years.

The prevalence of obesity is influenced by age, gender, race, and socioeconomic status. Obese population has been increasing in the United States in part because of an increasing number of old people. More than one-third (34.9%) of adults were obese in 2011–2012. In 2011–2012, the prevalence of obesity was higher among middle-aged adults (39.5%) than among younger (30.3%) or older (35.4%) adults (19).

This tendency has great effect on population health as a high BMI is linked to a higher morbidity and mortality rates (3, 20). It is also known that obesity has a negative influence on levels of testosterone and estrogens, as well as SHBG (21). However, the impact of obesity on FT is not so well-established (4, 5, 22). These hypogonadic patients suffer from androgen deficiency symptoms that can be relieved with androgen replacement therapy, leading to a better quality of life (23).

Free testosterone and FSH plays an important role in spermatogenesis (24). As exposed before, testosterone significantly decreases in obese men, but the decrement of free testosterone is not so prominent (25). It has been reported that obese men have a mean FT level only 5% lower than normal weight men (26). In addition, there is no evidence of a relationship between BMI and FSH. These findings strongly suggest that endocrine control of spermatogenesis is maintained even in obese men.

In a large review, eighteen of the twenty studies measuring testosterone and 15 of the 16 studies measuring SHBG reported negative relationships between BMI and these hormones. Of 12 studies that investigated free testosterone, 10 reported a negative relationship with BMI (27). Our study reported a negative relationship between BMI with TT, FT and SHBG. In the other hand, no association between FSH and LH with BMI was observed.

Regarding the relationship between BMI and SHBG levels, the negative correlation reported in several studies could be explained by a reduced hepatic globulin synthesis due to inhibition by excessive circulating insulin in men with a higher BMI (4), reduction observed in our group of patients.

Varicoceles are diagnosed in up to 15% of the general population (28), in about one-third of men who present for evaluation of primary infertility and in up to 40% of patients with infertility (29). Our study revealed an increased prevalence of varicocele in elderly men (36.3%) compared to general population.

Moreover, recent studies have indicated that varicocele has a detrimental effect on testicular Leydig cell function, reducing serum testosterone levels (30, 31). A higher prevalence of varicocele and a lower serum level of TT and FT have been correlated to aging (32). Based on these evidences, we can assume that men with varicocele may be at risk for premature androgen deficiency and that varicocele repair may reduce this risk. A recent study showed that microsurgical varicocele ligation resulted in a significant increase in serum testosterone levels in more than two-thirds of men (14). In addition, some studies have shown that BMI has a protective effect for the development of varicocele (33, 34). However, there seems to be an inverse relation between BMI and serum levels of testosterone, and varicocele could be involved in this relationship. Despite evidences suggesting that varicocele may have an impact on testicular function, in our studied population, it was not observed. Maybe the reduced number of patients with grade 3 varicocele included in the study compared to previous reports (14) can explain it. To our knowledge, no previous study has reported the relationship of ageing, obesity and varicocele.

Our study has some limitations that should be mentioned. It is a cross sectional study, thus patients were not followed over time. The real impact of aging or varicocele in each subject cannot be assessed due to our study design. In addition, the levels of estradiol were not assessed. These data could explain the impact of the BMI on hormone profile. Also, we were not able to determine if weight loss could improve the hormone profiles of obese patients. We believe that the low number of obese patients with clinical varicocele may have impacted on our results. This is a limitation of our sample characteristics.

## CONCLUSIONS

Men with higher BMI have lower serum levels of total testosterone, free testosterone and SHBG, which impair gonadal function. The presence of varicocele as well as its grade has no impact on hormone levels of elderly men.

## Abbreviations

KGRY: = Data acquisition and its interpretation. Drafted the manuscript
MC: = Study design and critical review for intellectual content
FCM: = Critical review for intellectual content. Drafted the manuscript
BCT: = Data acquisition and its analysis
RF: = Data acquisition and its analysis
GCF: = Study design
AAA: = Study design
MS: = Supervisor
